# Molecular and Cellular Mechanisms Modulating Trained Immunity by Various Cell Types in Response to Pathogen Encounter

**DOI:** 10.3389/fimmu.2021.745332

**Published:** 2021-10-04

**Authors:** Orlando A. Acevedo, Roslye V. Berrios, Linmar Rodríguez-Guilarte, Bastián Lillo-Dapremont, Alexis M. Kalergis

**Affiliations:** ^1^ Millennium Institute of Immunology and Immunotherapy, Departamento de Genética Molecular y Microbiología, Facultad de Ciencias Biológicas, Pontificia Universidad Católica de Chile, Santiago, Chile; ^2^ Departamento de Endocrinología, Facultad de Medicina, Pontificia Universidad Católica de Chile, Santiago, Chile

**Keywords:** trained immunity, unspecific cross-protection, epigenetics, metabolic reprogramming, innate memory

## Abstract

The induction of trained immunity represents an emerging concept defined as the ability of innate immune cells to acquire a memory phenotype, which is a typical hallmark of the adaptive response. Key points modulated during the establishment of trained immunity include epigenetic, metabolic and functional changes in different innate-immune and non-immune cells. Regarding to epigenetic changes, it has been described that long non-coding RNAs (LncRNAs) act as molecular scaffolds to allow the assembly of chromatin-remodeling complexes that catalyze epigenetic changes on chromatin. On the other hand, relevant metabolic changes that occur during this process include increased glycolytic rate and the accumulation of metabolites from the tricarboxylic acid (TCA) cycle, which subsequently regulate the activity of histone-modifying enzymes that ultimately drive epigenetic changes. Functional consequences of established trained immunity include enhanced cytokine production, increased antigen presentation and augmented antimicrobial responses. In this article, we will discuss the current knowledge regarding the ability of different cell subsets to acquire a trained immune phenotype and the molecular mechanisms involved in triggering such a response. This knowledge will be helpful for the development of broad-spectrum therapies against infectious diseases based on the modulation of epigenetic and metabolic cues regulating the development of trained immunity.

## Introduction

The immune system represents our main line of defense against infections and other diseases. For centuries, this type of response has been divided into two large branches: innate and adaptive immunity (1). The innate immune system represents the first barrier that aims to limit the ability of pathogens to spread through our body (2, 3). This response involves various innate cells including neutrophils, monocytes, macrophages, dendritic cells (DCs), Natural Killer cells (NK cells), as well as non-immune cells, such as the epithelium (4). The adaptive immune response corresponds to the second barrier of the immune system. Unlike the innate system, the adaptive response is antigen-specific and generates long-lasting protection, mainly mediated by T and B lymphocytes (1). It has been shown that effective memory immune responses rely on the interaction between cells of the innate and adaptive immune cells (5, 6). While activation of innate immunity provides the first line of defense against infections, it also primes the adaptive immune response *via* antigen presentation and cytokine production (7–10).

Furthermore, adaptive immunity can enhance the antimicrobial machinery of innate cells, making them more effective at clearing pathogenic microorganisms (11, 12). An additional layer of complexity is added to this network of interactions after recent findings showing the ability of innate cells to adopt a memory phenotype upon encountering different kinds of stimuli derived from pathogens (13, 14). During the last decade, such observations led to the establishment of the concept of “trained immunity”, which modified the traditional conception of memory responses that only used to apply to adaptive immunity (15). This new evidence suggested that innate-immune cells can adopt a memory-like phenotype through different epigenetic, metabolic and functional changes (16, 17). Furthermore, it has been proposed that non-immune cells can develop some of the features of this memory-like phenotype (18–20). Trained immunity can be triggered by a wide range of stimuli, including the bacteria Bacillus Calmette Guerin (BCG), β-glucan (a fungal cell wall component) and sex-related hormones, such as β-estradiol (19, 21, 22). Notably, the capacity to induce trained immunity is not only restricted to microbial-derived signals and hormones, as other endogenous ligands such as oxidized low-density lipoproteins (oxLDL) can also contribute to initiating this type of response (23). In the current article, we will summarize the mechanism underlying the development of trained immunity, the cells able to develop this response, and their contribution to controlling infectious diseases.

## Mechanisms Underlying the Establishment of Trained Immunity

Epigenetic changes on histones that interact with the DNA are one of the fundamental factors for the establishment of trained immunity (24). These epigenetic modifications include changes in histone methylation, which may promote or repress gene transcription (25). Recent studies underscore the contribution of long non-coding RNAs (LncRNAs) in triggering trained immunity due to their ability to promote chromatin remodeling by a direct interaction with chromatin while allowing the assembly of histone-modifying enzymes (26). The 3D arrangement of chromatin and proteins associated during this process occurs in discrete regions of enriched chromosomal contacts known as topologically associated domains (TADs) (27). Within TADs, genes with related functions are brought into proximity through the formation of chromosomal loops, which facilitate clustered regulation of gene transcription (27). A recent study described a novel class of LncRNAs, known as Immune-gene Priming LncRNAs (IPLs) involved in accumulating H3K4me3 at the promoters of trained immune genes (26). Bioinformatic analyses revealed the presence of a single LncRNA associated with TADs in which trained immune transcripts interacted with the histone H3Lys4 methyltransferase (MLL1) to direct local H3K4me3 accumulation (26). The IPL found in this study corresponds to UMLILO (upstream master LncRNA of the inflammatory chemokine locus) and was shown to regulate gene expression in TADs containing the genes encoding for IL8, CXCL1, CXCL2, and CXCL3 on human monocytes (26). This study also described two other important points. First, in mice, the TAD that contains these chemokines lacks UMLILO, therefore the expression of these genes cannot be trained (26). Of note, the insertion of UMLILO in the TAD of murine macrophages comprising these chemokines resulted in the training of such genes. These observations support the notion that LncRNA-mediated regulation is essential in establishing trained immunity (26). Secondly, genetic ablation of UMLILO in human monocytes abrogates the induction of trained immunity in these cells, further supporting the critical role of LncRNA in promoting innate immune training (26). In conclusion, targeting LncRNA appears as an attractive target for modulating the establishment of trained immunity and regulating inflammation (26).

The development of trained immunity also involves metabolic changes that ultimately lead to enhanced cytokine responses (28). Studies performed in mice highlight the ability of *C. albicans* infection in conferring protection against *S. aureus* (21, 29, 30). *In vitro* studies showed that trained immunity induced by *C. albicans* is mediated by the cell wall component β-glucan, which induces monocyte epigenetic remodeling and functional reprogramming (21, 30). In this case, trained monocytes accumulate the metabolite fumarate produced during the tricarboxylic acid cycle (TCA) (31). Fumarate then binds and inhibits histone demethylase 5 (KDM5) activity involved in the demethylation of H3K4 (32). Under this scenario, fumarate accumulation increases H3K4 tri-methylation in the promoters of genes encoding pro-inflammatory cytokines TNF-α and IL-6 (32). Different studies have been carried out to understand the interplay between metabolites and histone-modifying enzymes involved in establishing trained immunity. One example is acetyl-CoA, which is fundamental for the activity of histone acetyltransferases (HATs) (33, 34). Evidence showed that increased activity of metabolic pathways leading to acetyl-CoA production leads to an increased frequency of acetylation marks on histone tails (35). In mammalian cells, these changes are dependent on adenosine triphosphate (ATP)-citrate lyase (ACLY), which converts citrate into acetyl-CoA (36). Therefore, substrates that can be converted into citrate, such as glucose, fatty acids or glutamine, can ultimately lead to ACLY-dependent acetylation of histones (33). Another metabolite modulating trained immunity is itaconate, a derivative from the TCA cycle recognized by the ability to form adducts with glutathione (GSH) (37). Oxidized GSH inhibits the activity of S-adenosyl methionine synthetase, MAT1A involved in the synthesis of s-adenosyl methionine (SAM), the primary substrate of histone methyltransferases (HMTs) which are also modulators of trained immunity (38, 39). In the worm *C. elegans*, low SAM concentration restricts H3K4me3 accumulation at immune-responsive promoters, limiting the expression of genes necessary for the innate immune response against bacterial infection (40).

Another essential change observed in β-glucan- and BCG-trained cells is the increased ratio of nicotinamide adenine dinucleotide (NAD^+^) over the reduced form (NADH) (41). NAD^+^ is a required cofactor for the activity of de-acetylating enzymes known as sirtuins (SIRTs) (42). These enzymes catalyze the removal of lysine acetyl groups from different proteins, including histones (42). By removing acetyl groups, lysine residues of histones recover their positive charge and become more tightly bound to DNA leading to inhibition of gene transcription (43). During the establishment of trained immunity, a higher ratio of NAD^+^ over NADH promotes the activity of SIRTs, which subsequently influence inflammatory responses (44). *In vivo* studies showed that mice lacking SIRT2 displayed enhanced pro-inflammatory responses in a model of colitis induced by dextran sulfate sodium (DSS) as compared to wild-type mice (44). In this case, SIRT2 deficiency leads to the increased polarization of macrophages toward a pro-inflammatory phenotype (44). Thus, therapies targeting SIRT2 on macrophages could be explored to treat colitis (44). In addition, activity of Sirtuins also represses the expression of genes involved in glycolytic metabolism, including the transcriptional regulator HIF1α, a pivotal modulator for the induction of trained immunity (41). Studies related to other factors that trigger trained immunity showed that administration of BCG vaccine on healthy human volunteers up-regulates the production of IL-6 by monocytes and neutrophils upon exposure to *S. aureus* (45, 46). However, it is still not fully understood how these complex interactions take place in different immune and non-immune cells. The following sections will focus on the currently known drivers of trained immunity on different innate-immune and other non-immune cells and their contribution during infectious diseases.

## Trained Immunity in Neutrophils

Circulating human neutrophils are the most prominent immune cells present in the blood (47). These cells are characterized by their short lifespan (6–10 h) and their rapid recruitment following BCG or *Mycobacterium tuberculosis* (*M. tuberculosis*) infection (48, 49). *In vitro* studies indicate that neutrophils derived from BCG-vaccinated individuals showed a trained immunity phenotype (46, 50). It has been suggested that such phenotype on neutrophils relies on the ability of BCG to train hematopoietic bone marrow stem cells precursors (HSCPs), which subsequently can differentiate into neutrophils (19). In addition, intravenous rather than subcutaneous immunization of mice with BCG results in trained immunity on neutrophils. Such differences may be explained by the access of BCG to the bone marrow through blood circulation (19). Trained immunity induced by BCG on neutrophils is characterized by increased expression of CD11b and Interleukin-8 (IL-8) following re-stimulation with unrelated BCG stimuli, such as *S. aureus* or lipopolysaccharide (LPS) (46). Since both markers were involved in neutrophil activation and chemotaxis, respectively (51), these data suggest that trained immunity induced by BCG on neutrophils promotes neutrophil recruitment and activation, which is also essential for bacterial clearance (52).

Studies using mice vaccinated with BCG *via* the intranasal route showed that neutrophils accumulate in the lungs as early as 1 to 3 days post-inoculation of BCG (29). Interestingly, these recruited neutrophils showed the ability to kill *M. tuberculosis*, supporting a role of BCG in promoting neutrophil antimicrobial responses (49). Furthermore, studies in the mouse model showed that neutrophil depletion before BCG vaccination resulted in increased bacterial loads compared to isotype control-treated mice (50). These data suggest that neutrophils play a significant role in reducing the mycobacterial burden and are necessary for the protection conferred by BCG vaccination (50). Further studies are needed to determine if trained immunity on neutrophils modulates the production of chemokines important to attract other immune cells, which might complement neutrophil-mediated responses.

*In vitro* studies of human-derived neutrophils indicate that BCG vaccination increases reactive oxygen species (ROS) production by these cells upon *C*. *albicans* stimulation as compared to neutrophils from non-vaccinated individuals (46). In addition, neutrophils derived from BCG-vaccinated subjects showed a higher production of lactate and enhanced killing activity against *C. albicans* in comparison to neutrophils from the non-vaccinated subjects (46). These data suggest that the development of trained immunity induced by BCG is associated with increased glycolytic activity and favors neutrophil-mediated killing of *C. albicans and M.* tuberculosis (46, 50). These results raise new questions, such as the way trained neutrophils may affect the function of other cell types. The contribution of non-trained neutrophils modulating the function of neighboring cells, such as macrophages and T lymphocytes has been documented (53, 54). Therefore, it would be essential to examine whether BCG-trained neutrophils may regulate the responses displayed by these immune cells. Neutrophils have been shown to train macrophages to acquire a long-lasting enhanced protective phenotype against infection (54). Furthermore, it is reported that neutrophils can activate T cells through antigen presentation (55). However, further studies at the single-cell level are needed to elucidate whether the transcriptional landscape of trained neutrophils is present on a particular subset of neutrophils or involves this entire cell population.

## Trained Immunity in Monocytes and Macrophages: General Features

Monocytes are part of other subset of myeloid cells responsible for producing pro-inflammatory cytokines during an infection (56). These cells circulate in the bloodstream for up to 3 to 5 days, from where they then differentiate into macrophages (57). Monocytes and macrophages are mononuclear phagocytes that mediate fundamental innate immune processes such as pathogen clearance, inflammatory cytokine production, and tissue repair (58, 59). The ability of monocytes and macrophages to adopt a trained immunity phenotype is an active matter of study (60). Epigenetic changes, such as H3K4me3 were elevated in promoters of genes encoding for pro-inflammatory cytokines after stimulation of monocytes with BCG or β-glucan (61, 62). This notion is supported by the observation that inhibition of histone methyltransferases using 5′-Deoxy-5′-methylthioadenosine (MTA) suppressed monocyte training by *C. albicans* or β-glucan. These data provide additional basis for the role of histone methylation in the training of monocytes (63). H3K4me3 is significantly increased at the Toll-like receptor 4 (TLR4) level in circulating monocytes collected after BCG vaccination as compared to values obtained from monocytes isolated before BCG vaccination (64). In addition to the activation of the Toll-like receptor (TLR) signaling pathway (63), immune training by β-glucan is dependent on the Dectin-1/Raf-1 pathway (65). The interaction between monocytes and β-glucan through Dectin-1 activates the spleen tyrosine kinase and the caspase recruitment domain-containing protein 9 (Syk/CARD9), resulting in the activation of the transcription factor NF-κB (61, 66). The inhibition of Dectin-1 by laminarin in purified peripheral blood monocytes from healthy donors suppressed β-glucan-induced trained immunity (63). These findings suggest that Dectin-1 is a significant driver of trained immunity in monocytes (63).

## Metabolic Pathways Involved in the Training of Monocytes and Macrophages

Different metabolic pathways are involved in the regulation and development of trained immunity in monocytes (32, 67, 68). Trained monocytes show high glucose consumption, high lactate production, and a high ratio of nicotinamide dinucleotide and reduced adenine (NADH), reflecting a change in metabolism with increased glycolysis (68). These changes depend on signaling through Akt, mTOR (mammalian target of rapamycin), and HIF-1α (hypoxia-inducible factor 1α) (60). In this sense, priming of monocytes with BCG increases the phosphorylation of Akt (68). Inhibition of Akt by Wortmannin during the first 24 hours of training with BCG prevents the increase in the production of cytokines by re-stimulation with LPS (**Table 1**) (67, 68). Inhibition of mTOR by rapamycin leads to similar effects inhibiting the production of TNF-α and IL-6 following re-stimulation of cells with LPS (60) and pre-treatment of cells with ascorbate that inhibits the HIF-1α pathway (**Table 1**) (60). Treatment of cells with metformin or 2-deoxy-glucose abrogates enhanced cytokine production by inhibiting hexokinase-2 (37). Furthermore, inhibition of glycolytic pathways inhibited epigenetic modifications in the promoters of genes encoding IL-6 and TNF-α (**Table 1**) (67). The increased glycolysis observed in trained monocytes promotes the accumulation of fumarate, which inhibits histone demethylase 5 KDM5. Therefore favoring H3K4me3 on the promoters of pro-inflammatory cytokines TNFα and IL-6 (32). Other metabolic pathways involved in the development of trained immunity include the synthesis of cholesterol, which can be inhibited by statins (**Table 1**) (30), which then prevent the enrichment of H3K4me3 in the promoters of genes that encode IL-6 and TNF-α (32, 67, 68). In conclusion, several metabolic pathways could be targeted to increase trained immunity and enhance the mechanisms of immune defense against infections.

**Table 1:** Inhibitors of different signaling, metabolic and epigenetic changes are involved in inducing trained immunity against infectious diseases.

Inhibitors of signaling pathways			
Cell type	Inhibitor	Function	Reference
Monocytes	Rapamycin	mTOR inhibitor	(23)
	Wortmannin	Akt inhibitor	
	Ascorbate	HIF-1α inhibitor	
	Metformin	AMPK inhibition	
**Inhibitors of metabolic pathways**			
**Cell type**	**Inhibitor**	**Function**	**Reference**
Monocytes	2-Deoxy Glucose	Inhibits Hexokinase 2	(68)
**Inhibitors of epigenetic modifiers**			
**Cell type**	**Inhibitor**	**Function**	**Reference**
Macrophages	MTA	Methyltransferase inhibitor	(63)
Bronchial epithelial cells	Epigallocatechin-3-gallate (EGCG)	Inhibition of histone acetyltransferase	(20)
	BIX01294	Inhibitor of histone Methyltransferase	

During the differentiation of monocytes into macrophages, training induced by β-glucan increases the expression of genes involved in metabolic and inflammatory pathways, and such changes are dependent on cAMP signaling. In this line, cAMP inhibitors including 2 ′, 5′-dideoxyadenosine and propranolol can prevent the increased production of IL-6 and TNF-α induced by β-glucan training (69). Additionally, monocytes and macrophages exposed to β-glucan showed a trained immune phenotype dependent on the metabolism of glutathione, a relevant antioxidant molecule involved in detoxifying free radicals (70). Along these lines, plasma concentration of IL-1β from BCG-vaccinated individuals are positively associated with serum glutathione concentrations (71). Furthermore, trained immunity also up-regulates the expression of genes involved in glutathione metabolism, suggesting an increase in glutathione synthesis and a higher glutathione recycling rate (71). Finally, single nucleotide polymorphisms (SNPs) in these genes are associated with changes in pro-inflammatory cytokine production after *in vitro* training by β-glucan and BCG (71). Therefore, enzymes whose activity is dependent on cAMP or glutathione could be used as novel targets to modulate trained immunity.

## Hormonal Control of Trained Immunity Responses in Monocytes and Macrophages

Studies *in vivo* have shown that administration of β-glucan in mice attenuates the hallmarks of sepsis-induced by *Escherichia coli* infection in a sex-dependent manner (22). In this regard, β-glucan mediated prevention of lung injury by the induction of trained immunity worked better in females than in males (22). Interestingly, this work showed that female hormones, such as estrogens are involved in the development of trained immunity, which can also explain the increased susceptibility of male over female mice to *E. coli*-induced sepsis (22). Mechanistically the authors showed that exposure of macrophages to β-estradiol, which is a form of the female hormone estrogen (72), polarizes these cells toward a pro-inflammatory M1 phenotype with enhanced ability to kill *E. coli* and therefore more efficient at preventing sepsis (22). Finally, this study also showed that treatment of macrophages with β-estradiol inhibited the nuclear translocation of RelB, a member of the non-canonical pathway of NF-κB, which contributes to macrophage polarization towards the M1 pro-inflammatory phenotype (73).

Remarkably, the role of estradiol and sex-depended hormones in trained immunity remains controversial. *In vitro* studies have shown that sex hormones such as estradiol and dihydrotestosterone (DHT) can inhibit the production of pro-inflammatory cytokines during the trained immune response elicited by BCG (48). Therefore, further research is required to define the specific contribution of sex hormones to trained immunity and how differs from the induction by BCG or β-glucan. To our knowledge, there is only limited studies comparing the metabolic and epigenetic landscape associated with immune training induced by BCG in comparison to β-glucan (67, 74, 75).

## Trained Immunity on Alveolar Macrophages and Involvement of Resident Cells

Alveolar macrophages (AMs) are the main sentinels that reside in the alveolar space and represent an example of tissue-resident cells in which trained immunity has been described (76). Most of the current knowledge in innate immune memory comes from systemic infection or immunization data, which induces innate memory in circulating monocytes or macrophages (21, 62). The establishment of trained immunity on AMs provides an example for the involvement of adaptive immunity for the development of trained immunity on innate immune cells (76). Consistently with this notion, a recent study showed that the interaction between alveolar macrophages (AMs) and T cells in the surface mucosal allows the development a memory-like response in macrophages (76).

Evidence showed that *S. pneumoniae* infection following adenovirus vaccination induces trained immunity on AMs *via* a rapid increase of chemokines and neutrophilia (76). In this process, CD8^+^ T cells are required for the priming of AMs through secretion of IFN-γ (76). Following infection, AMs up-regulate the expression of MHC II (76). Furthermore, when CD8^+^ T cells were depleted, a loss of AMs memory was observed at 7 and 28 days post depletion, accompanied by a decrease in AMs glycolytic rate (76). Although this type of interaction between an innate and adaptive immune response generates trained immunity phenotype in AMs, it would be important to evaluate whether other resident cell populations, such as DCs can be trained in this manner (76).

## Trained Immunity in NK Cells

**Figure 1:**
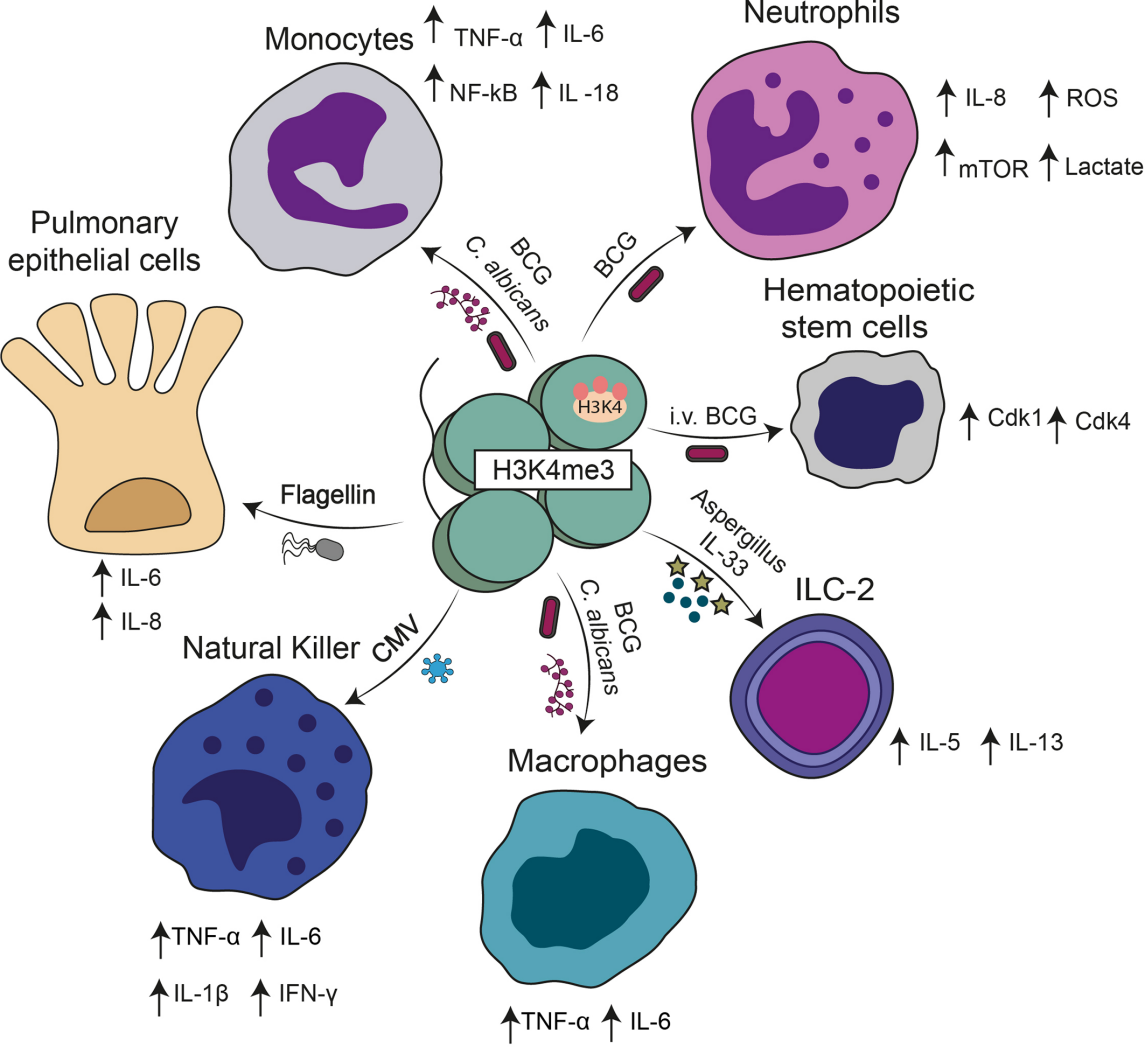
Cell subsets in which trained immunity has been described. Different stimuli including BCG, β-glucan, cytokines, CMV, and bacterial components can induce a trained immunity phenotype. A common hallmark of trained immunity in these cases is the presence of H3K4me3 in the promoters of genes encoding for different cytokines described in the figure.

Natural killer (NK) cells are another cell type with the ability to adopt an immune memory-like phenotype for viral pathogens (73). Consistently with this notion, it was shown that NK cells can adopt a memory phenotype against murine cytomegalovirus (MCMV) (77). Studies in mice showed that adoptive transfer of MCMV-induced memory NK cells significantly increased the survival of newborn mice upon MCMV infection as compared to mice transferred with unexperienced NK cells (78). The mechanisms underlying trained immunity, in this case, involved structural changes at the chromatin structure, in which the suppressive DNA methylation is reduced in the locus of genes codifying for antiviral cytokines such as interferon (IFN)-γ (73). Furthermore, regulatory genes important for cell activation become accessible for the transcriptional machinery allowing a faster response upon stimulation (73). Studies from cohort patients showed that cytomegalovirus (CMV) seropositivity was associated with the expansion of memory NK cells (79). Identifying such memory cells was based on the expression of the activating receptor NKG2C, which recognizes MHC-I presented peptides leading to cell activation (80). NK memory-like cells have also been shown to be induced by the BCG vaccine (81). In humans, enhanced IFN-γ production by NK cells from vaccinated volunteers was still present over one year after vaccination, suggesting that BCG induces long-lasting memory in NK cells (81). Furthermore, this BCG-induced memory increased production of IFN-γ, IL-1β, IL-6, and TNF-α following challenges with *M. tuberculosis* and *M. tuberculosis*-unrelated pathogens, such as *C. albicans* and *S. aureus* (**Figure 1**) (81).

Experimental studies have shown that cytokine priming with an antibody cocktail containing IL-12, IL-18, and IL-15 is sufficient to program NK cells to produce higher levels of IFN-γ upon re-challenge with cytokines or antibodies targeting activating receptors, such as Ly49H and NK1.1 (82). Furthermore, the ability to produce IFN-γ is maintained at least for a month and passed from mother to daughter cells, suggesting that this memory is epigenetically controlled (66). This notion is further supported by observation that the pre-activation of NK cells with IL-12, IL-18, and IL-15 cytokines promoted demethylation of IFN-γ regulatory elements (83). These findings suggest that NK cells can develop non-antigen-specific memory, in a process driven by chromatin remodeling (82, 83).

## Trained Immunity in Innate Lymphoid Cells

Innate lymphoid cells (ILCs) display classical lymphoid cell morphology lacking the diversified antigen receptors expressed on T and B cells (84). ILCs consist of three groups: group 1 ILC (ILC1) producing IFN -γ, group 2 ILC (ILC2) producing IL-4, IL-5, and IL-13, and group 3 ILC (ILC3) that produce IL-17 and IL-22, which have functions similar to their pairs of adaptive immunity, helper T cells (Th) of the type Th1, Th2, and Th17 respectively (84, 85). Thus, the cytokines produced by ILCs contribute to multiple immune pathways, including lymphoid development, metabolic homeostasis, maintenance of appropriate immune responses to commensals and pathogens in mucosal barriers, enhancing adaptive immunity, and regulating tissue inflammation (84, 85).

During infections of humans or mice with *M. tuberculosis*, ILCs are decreased in peripheral blood and migrate to the site of infection in which recruitment is regulated through the CXCL13/CXCR5 axis (86). The IL-22 produced by ILC3 is essential to inhibit excess inflammation and damage to epithelial cells in mice infected by *M. tuberculosis*; these cells also reduce the bacterial load (87). It has recently been shown that ILCs have immune responses that resemble the training observed in other cells of the innate immune system; intranasal injection of BCG can increase the recruitment of ILCs to the lungs and improve IFN-γ production (13). However, it was also documented that exposure of ILC-2 to allergens such as *Aspergillus* induces a pathological trained immunity response characterized by the secretion of Th2 related cytokines such as IL-5 and IL-13 (**Figure 1**) (88). These data suggest that trained immunity can also generate pathological responses depending on the stimuli involved. Further studies are needed to elucidate the epigenetic changes and metabolic factors associated with ILC-2 training (88).

## Trained Immunity on Hematopoietic Stem Cells

Hematopoietic stem cells (HSCs) are long-lived cells mainly present in the bone marrow (BM), which can self-renew and generate multipotent and lineage-committed hematopoietic progenitors, which then originate the entire set of cells present in the mammalian blood system (89). Interestingly, a recent study showed that allowing the access of BCG vaccine to the bone marrow employing intravenous immunization rather than subcutaneous (sc) route in mice modified the transcriptomic landscape of HSCs resulting in enhanced myelopoiesis (19). As compared to the standard subcutaneous route, an intravenous administration of BCG favors the expansion of HSC progenitors and the up-regulation of different genes involved in DNA replication, cell division, and cell cycle (19). Among them, various key regulators of cell cycle progression such as Cdk1, Cdk4, and other cyclins were strongly up-regulated in HSCs of mice vaccinated intravenously with BCG as compared with HSCs from animals immunized subcutaneously BCG (**Figure 1**) (19). Interestingly, macrophages derived from the bone marrow of mice immunized intravenously with BCG, but not subcutaneously, showed significantly better protection against an *in vitro M. tuberculosis* challenge (19). Therefore, the outcomes of trained immunity also involved changes in the precursors of innate cells, such as macrophages and neutrophils (19). In the latter case, as mature neutrophils have a short lifespan it was demonstrated that trained immunity can act *via* the modulation of hematopoietic stem cells (HSCs) (90). In this *in vivo* study, intraperitoneal injection of mice with β-glucan increased the numbers and frequency of multipotent progenitors and hematopoietic progenitors in the bone marrow and led to enhanced cell-cycle progression in HSCs (90). This was a beneficial response facing a second heterologous challenge with LPS or chemotherapy-induced myelosuppression (90). However, elevated production of cytokines, such as IFN-γ can also produce unwanted cell survival effects because sustained IFN-γ signaling can have negative consequences on hematopoietic stem cells by increasing susceptibility for secondary stress-induced apoptosis (91). However, is still controversial whether IFN-γ alone induces HSC apoptosis. *In vitro* IFN-γ treatment of human HSCs co-cultured with stromal cells augmented HSC apoptosis (92). In addition, RNA expression studies of HSCs from patients with high IFN-γ levels have indicated an increase in the transcription of apoptosis-related genes (93). Furthermore, stimulation of HSCs with IFN-γ alone showed no increase in apoptosis (94). Therefore, suggesting that interaction of IFN-γ with the action of other cells modulates HSCs apoptosis. These findings provide valuable information to developing new therapeutic approaches to target trained immunity and cytokine production for diseases in which cell cycle disorders play a significant role, such as cancer (95).

## Trained Immunity in Bronchial Epithelial Cells

Although many reports have shown that trained immunity is triggered in innate immune cells, a recent study highlights the ability of respiratory epithelial cells in acquiring a memory phenotype after exposure to flagellin from *Pseudomonas aeuroginosa* (*P. aeuroginosa*) (20). Specifically, *in vitro* studies showed that pre-exposure of human bronchial epithelial cells (BEAS2-B) to this bacterial component increases their inflammatory response to living conidia from *Aspergillus fumigatus* (*A. fumigatus*) and LPS (20). In this case, trained cells produced increased levels of IL-8 and IL-6 following LPS or *A. fumigatus* challenge in comparison to non-trained controls (**Figure 1**). Trained immune responses were shown to rely on epigenetic modifications. For example, inhibition of histone acetyltransferase with epigallocatechin-3-gallate (EGCG) significantly reduced the flagellin-induced IL-8 trained immune response to *A. fumigates* (**Table 1**) (20). Similarly, treatment of cells with BIX01294, an inhibitor of histone methyltransferase which prevents methylation of H3K4, also reduced flagellin-induced IL-8 trained immune response without affecting the IL-8 levels observed in non-trained cells (**Table 1**) (20).

## Trained Immunity in Skin Stem Cells

Skin stem cells have been also shown capable of generating a prolonged memory to acute inflammation, which allows accelerating the restoration after subsequent damage in a model of skin inflammation induced by TLR7 and the NALP3 agonist imiquimod (96). Sequence analyses revealed an increase of inflammation and hyper proliferation-associated pathways, including apoptosis signaling, interleukin signaling, oxidative stress response, and PI3 kinase pathways (96). It was suggested that the memory experienced by the inflammation of skin epithelial stem cells may be the basis for the recurrent skin inflammation exhibited by patients with autoimmune disorders, such as psoriasis and atopic dermatitis, as well as hyperproliferative disorders, including cancer (96).

## Trained Immunity in the Gastrointestinal Tract

Evidence from recent studies showed that β-glucan can also influence intestinal inflammation and epithelial barrier function. Experiments in mice showed that oral administration of β-glucan could aggravate intestinal inflammation in a model of dextran sodium sulfate (DSS)-induced colitis (97). In addition, mice lacking dectin-1, the receptor for β-glucan, also showed augmented susceptibility to DSS-induced colitis, a finding recapitulated in humans with specific polymorphisms in dectin-1 (97). Prolonged oral treatment of mice with antifungals increases disease severity in models of chronic colitis and chronic allergic airways disease (98). Such findings highlight the importance of a healthy fungal community in gut homeostasis. Furthermore, these results also suggest that gut microbiota may influence peripheral immune responses and pulmonary allergies. In this line, additional research is needed to further elucidate the role of trained immunity in the gut in health and disease.

## Immunity Training in Against Protozoan-Mediated Pathologies

The trained immunity also confers protection against protozoan infectious agents, as demonstrated for Leishmaniasis, which is associated with a pro-inflammatory activity in monocytes and macrophages (99–102). A recent study shows that the induction of trained immunity by β-glucan increases the efficiency of phagocytosis and the clearance of *L. braziliensis*, in parallel with increased production of cytokines, specifically IL-6 and IL-10 (100). Such an increased immune response depends on the enhanced expression of IL-32 that induces antimicrobial peptides (100).

## Trained Immunity in Non-Infectious Pathologies

The trained immunity induced by BCG or β-glucan not only confers non-specific protection against infectious agents, but also to other pathologies, such as cancer (39). For example, the BCG vaccine can contribute to the anti-tumor immune response as a treatment in bladder cancer (39). The anti-tumor effect of BCG seem to rely on the ability to induce trained immunity in monocytes in which autophagy plays an essential regulatory role (103, 104). It has been shown during non-muscle-invasive bladder cancer that high expression of histone methyltransferase G9a is associated with poor cancer prognosis (39).The activity of this enzyme inhibits the induction of trained immunity in monocytes (39). In addition pharmacological inhibition of G9a improves trained immune responses, accompanied by a decrease in H3K9me2 marks on pro-inflammatory genes (39). Furthermore, *ex vivo* inhibition of G9a is associated with an amplified trained immune response and altered RNA expression of inflammatory genes in monocytes derived from patients suffering non-muscle-invasive bladder cancer (39).

In contrast, functional and transcriptional reprogramming toward a long-term pro-inflammatory phenotype of monocytes and macrophages after brief *in vitro* exposure to ox-LDL contributes to the progression to atherosclerosis (23, 105). Monocytes from patients with severe symptomatic coronary atherosclerosis display a pro-inflammatory phenotype associated with the epigenetic remodeling at the level of histone methylation and higher expression of speed-limiting enzymes of the glycolysis and pentose phosphate pathways (106). Consistently with this notion, bone marrow-derived and peritoneal macrophages from ApoE^-/-^ mice (a murine model of atherosclerosis) produced more pro-inflammatory cytokines after TLR stimulation by LPS than did saline-treated controls. These data suggest that an ApoE deficiency may lead to the development of trained immunity (107). However, additional research is needed to determine the relationship between trained immunity and this pathology.

## Concluding Remarks

While the induction of trained immunity has been shown for different types of innate cells, there is increasing evidence showing that other non-immune cells could also contribute to this type of immune/inflammatory response. Important questions that remain to be answered include elucidating the spectrum of cells that can develop a trained immunity phenotype and test if this process depends on the origin of cells. Finally, it will be important to elucidate the mechanism regulating trained immunity to provide an enhanced host defense while preventing a deleterious inflammation on different tissues. Answers to these questions in future studies are crucial to targeting trained immunity to develop broad-spectrum therapeutic approaches against infectious and non-infectious diseases.

## Future Perspectives

Here we have described and discussed as to how different epigenetic and metabolic changes can lead to the establishment of trained immunity. There is an intricate relationship between the metabolic reprogramming of cells and epigenetic changes given by the ability of multiple metabolites to modulate the activity of histone-modifying enzymes that subsequently regulate gene expression. However, many gaps of knowledge remain in this field. For example, it remains to define how long the changes associated to trained immunity last and if, in addition to epigenetic modulation, there are other post-translational modifications on proteins relevant for the induction of trained immunity. Finally, due to the wide arsenal of epigenetic and metabolic pathways involved in regulation of trained immunity there are several potential targets to modulate the magnitude of trained memory responses and subsequently regulate inflammation. However, because it is currently thought that epigenetic modulators may have pleiotropic unwanted effects, it is possible that using LncRNAs could constitute a more specific therapeutical approach. The knowledge about the factors controlling the folding state of a given LncRNA, as well as the identification of structural motifs involved in interaction with histone modifying enzymes, may contribute to the design of next-generation therapies able to increase the expression of relevant cytokines to enhance antimicrobial responses of different cell sub-sets.

## Author Contributions

OA, RB, BL-D, LR-G, and AK wrote the manuscript. AK reviewed the manuscript and approved the version to be published. All authors contributed to the article and approved the submitted version.

## Funding

This research was funded by CONICYT PAI project I781902009 Chile, as well as the Millennium Institute on Immunology and Immunotherapy grant number P09/016-F and ICN09_016. CORFO grant #13CTI-21526/P4 and P5; ANID/FONDECYT grants #3180570 (KB); #1190830 (AMK). Biomedical Research Consortium CTU06 (AK). COPEC-UC2019.R.1169. COPEC-UC2020.E.1.

## Conflict of Interest

The authors declare that the research was conducted in the absence of any commercial or financial relationships that could be construed as a potential conflict of interest.

## Publisher’s Note

All claims expressed in this article are solely those of the authors and do not necessarily represent those of their affiliated organizations, or those of the publisher, the editors and the reviewers. Any product that may be evaluated in this article, or claim that may be made by its manufacturer, is not guaranteed or endorsed by the publisher.
